# Predictors of Gleason score upgrading in patients with a biopsy diagnosis of grade group 1 prostate cancer

**DOI:** 10.55730/1300-0144.5962

**Published:** 2024-11-28

**Authors:** Barış ESEN, Bengi GÜRSES, Arif ÖZKAN, Mert KILIÇ, Yakup KORDAN, Tarık ESEN, Dilek ERTOY BAYDAR

**Affiliations:** 1 Department of Urology, Faculty of Medicine, Koç University, İstanbul, Turkiye; 2 Department of Radiology, Faculty of Medicine, Koç University, İstanbul, Turkiye; 3 Department of Urology, VKF AmericanHospital, İstanbul, Turkiye; 4 Department of Pathology, Faculty of Medicine, Koç University, İstanbul, Turkiye

**Keywords:** Prostate cancer, Gleason score, grade group 1, upgrade, multiparametric magnetic resonance imaging, phosphatase and tensin homolog, immunohistochemistry

## Abstract

**Background/aim:**

Transrectal ultrasound-guided biopsy is the most commonly used method in the diagnostic pathway of prostatic adenocarcinoma. The Gleason score of the tumor is the critical tissue-based determinant of patient management after diagnosis, and the main approach for low risk patients with grade group (GG) 1 disease is active surveillance rather than definitive interventions. However, a fair proportion of these cases are upgraded following radical prostatectomy (RP), if performed. We aimed to investigate the significance of clinicopathological parameters including phosphatase and tensin homolog (PTEN) protein in the prediction of higher final grade at RP in patients with a biopsy diagnosis of GG1 prostatic carcinoma.

**Materials and methods:**

Thirty-three patients who underwent robotic-assisted RP for grade GG1 disease at prostate biopsy were evaluated retrospectively. Their clinical, radiological, and pathological features were explored in addition to the final histological grade in RP. Upgrade was defined as any increase in Gleason score from the initial needle biopsy to pathological analysis of the entire surgical specimen. Expression of PTEN in prostate biopsy cores was evaluated through immunohistochemistry. Multivariate logistic regression was performed to detect independent predictors of tumor upgrading from biopsy to RP.

**Results:**

The final RP pathology revealed upgrading in 16 patients (48.5%) to GG2 disease. The statistics showed that Prostate Imaging Reporting and Data System score (≤3 vs. 4–5) of the index lesion and the number of involved cores in systematic biopsies (≤2 vs. >2) were the only independent predictors of the presence of a higher grade at RP (p < 0.05). The rate of PTEN loss for upgrading and nonupgrading patients was 25% and 5.9%, respectively, without statistical significance (p = 0.175).

**Conclusion:**

Multiparametric magnetic resonance imaging findings and number of tumor positive needle cores are useful parameters to apply for predicting higher grade disease in the RPs of patients with a GG1 tumor diagnosis after a prostate biopsy. Immunohistochemical PTEN analysis, on the other hand, does not provide significant information in this respect.

## 1. Introduction

Prostate cancer (PCa) is the second most commonly diagnosed cancer in men. Large radical prostatectomy (RP) series have previously documented that extraprostatic extension, seminal vesicle invasion, or lymph node metastasis is extremely rare in patients with grade group 1 (GG1) PCa [1]. Needle biopsy is the main way of diagnosing PCa, but unfortunately it does not always reflect the exact Gleason score of the tumor at RP. Pathological upgrading in RP is observed in 14%–51% of the cases diagnosed as GG1 disease at prostate biopsy [2]. Accurate prediction of grade rise in RP is of utmost importance to select the optimal treatment for patients in terms of active surveillance versus active treatment. Previously, several clinical and pathological parameters such as prostate-specific antigen (PSA), PSA density (PSAD), positive core percentage, and maximum percentage of cancer per core were reported as predictors of pathological upgrading [3]. The integration of multiparametric magnetic resonance imaging (mpMRI) into routine clinical practice resulted in higher accuracy in detecting GG ≥2 disease, and Prostate Imaging Reporting and Data System (PIRADS) score of index lesion (PIRADS ≤3 vs. >3) was found to be an independent predictor of higher grade and adverse pathology in initial GG1 cases by prostate biopsy [4]. Despite these promising results, upgrading of tumors after pathological examination of RP is still a common occurrence despite the extensive use of mpMRI today, indicating the need for additional biomarkers [4].

The loss of the protein encoded by the phosphatase and tensin homolog (*PTEN*) gene in PCa has been found to be associated with increased tumor grade and stage, earlier biochemical recurrence after RP, metastasis, and cancer-related mortality [5]. In some studies, PTEN loss detected by immunohistochemistry (IHC) was proposed to be a useful biomarker to predict pathological upgrading of GG1 diagnosis at prostate biopsy to ≥GG2 tumor in RP [6–8]. We have noted that these studies did not include findings of MRI as they were constructed before mpMRI entered the routine algorithm of PCa diagnosis. In our study, we aimed to explore whether immunohistochemical detection of PTEN loss can provide clinically significant data independent from mpMRI to predict RP upgrading in patients diagnosed with GG1 disease at the outset via prostate biopsy.

## 2. Materials and methods

### 2.1. Patients and procedures

**Figure 1:**
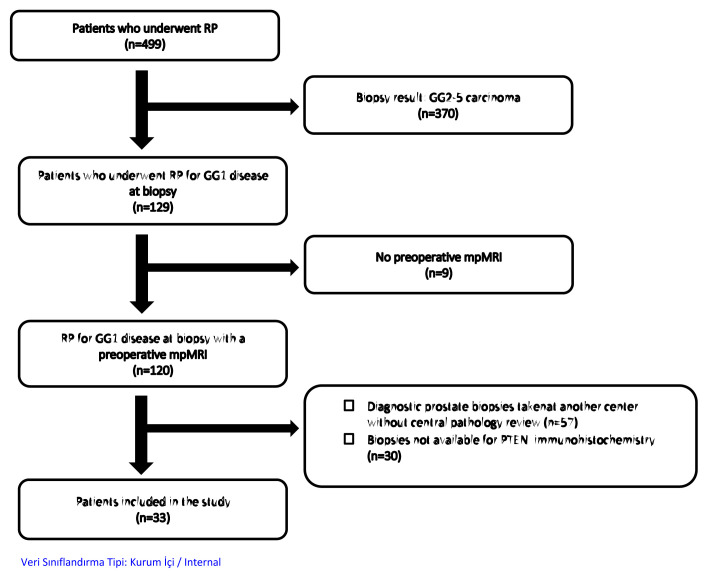
Flowchart for the construction of our study group.

After obtaining local ethics committee approval, we constituted our cohort from patients who underwent robot-assisted laparoscopic radical prostatectomy (RARP) after a diagnosis of GG1 PCa at prostate biopsy between 2018 and 2022. Thirty-three individuals met the inclusion criteria, which were:

- All prostatectomies were performed at our institution.
- Their diagnostic prostate biopsies were read either primarily (n = 29) or after submission for central review (n = 4) by an expert uropathologist in our hospital (Figure 1).
- We required the presence of mpMRI within 8 weeks before biopsy for each man.

Each prostate mpMRI scan was performed with a 3-T device (Magnetom Skyra; Siemens AG, Munich, Germany) using a 16-channel pelvic phased array coil at our center. External scans were checked for image quality and it was verified whether they were optimal for accurate PIRADS score determination. The PIRADS scoring system (version 2 and 2.1) was utilized to classify prostatic lesions in all cases. Each scan was evaluated only by a member from our specialized uroradiology team, who consisted of 4 radiologists highly experienced in the PIRADS system. The prostate biopsy techniques performed were MRI/US fusion biopsy (which targeted all PIRADS ≥3 lesions in addition to 12-core systematic biopsy) in 24 cases, real-time MRI-guided in-bore biopsy in 4 cases, and 12-core systematic-only biopsy in the remaining 5 patients. In the patients who underwent in-bore biopsy, 3 to 5 targeted tissue cores were obtained only from the lesion without systematic sampling. Clinical, pathological, and mpMRI-related parameters were recorded for each case. RARP was performed using da Vinci Si or Xi robotic systems (Intuitive Surgical Incorporation, Sunnyvale, CA, USA). Any GG2 or higher disease at RP was considered pathological upgrading. PTEN protein expression in the tumor samples found in the prostate biopsies was evaluated through standardized IHC in all patients.

### 2.2. Immunohistochemistry

The PTEN assay was performed on the BenchMark ULTRA (Roche Diagnostics, Switzerland) automated staining platform as previously described [9] with minute modifications in incubation temperatures. Briefly, 4-μm FFPE tissue sections were deparaffinized and heat-pretreated with EDTA to achieve antigen retrieval for 48 min at 100 °C. Slides were incubated with anti-PTEN rabbit monoclonal antibody [1:100 dilution; clone D4.3 XP, Cell Signaling Technology, Danvers, MA, USA] for 36 min at 37 °C. The protein was visualized using an OptiView DAB IHC Detection Kit (Ventana Medical Systems, Inc., Oro Valley, AZ, USA). Cases were considered to have PTEN protein loss if the intensity of cytoplasmic and nuclear staining was markedly decreased or entirely negative across >10% of malignant cells in comparison to represented benign prostatic glands and/or stroma.

### 2.3. Statistics

Statistical analysis was performed using SPSS version 24 (SPSS Inc., Chicago, IL, USA). Descriptive statistics were provided using either mean and standard deviation (SD) or median and interquartile range (IQR) for continuous parameters and percentages for categorical parameters. Univariate and multivariate logistic regression analyses were performed to detect independent predictors of tumor upgrading. The results were presented with odds ratios and 95% confidence intervals. A p-value of less than 0.05 was considered statistically significant.

## 3. Results

The mean patient age at prostate biopsy was 63.2 ± 8.0 years. The median serum PSA and PSA density (PSAD) were 5.1 ng/mL (IQR: 4.5–8.0) and 0.10 (IQR: 0.07–0.14), respectively.

The PIRADS score of index lesion was PIRADS <3 in 4 patients, PIRADS-3 in 10, PIRADS-4 in 15, and PIRADS-5 in 4. The median interval between prostate biopsy and RP was 6.9 weeks (IQR: 5.3–10.1). The final RP pathology revealed upgrading in 16 patients (48.5%) and this was a rise to GG2 disease in all of them. The upgrading rates were associated with increasing PIRADS scores, and these were 0%, 30%, 66.7%, and 75% for PIRADS <3, PIRADS-3, PIRADS-4, and PIRADS-5 index lesions, respectively (p = 0.041).

**Figure 2:**
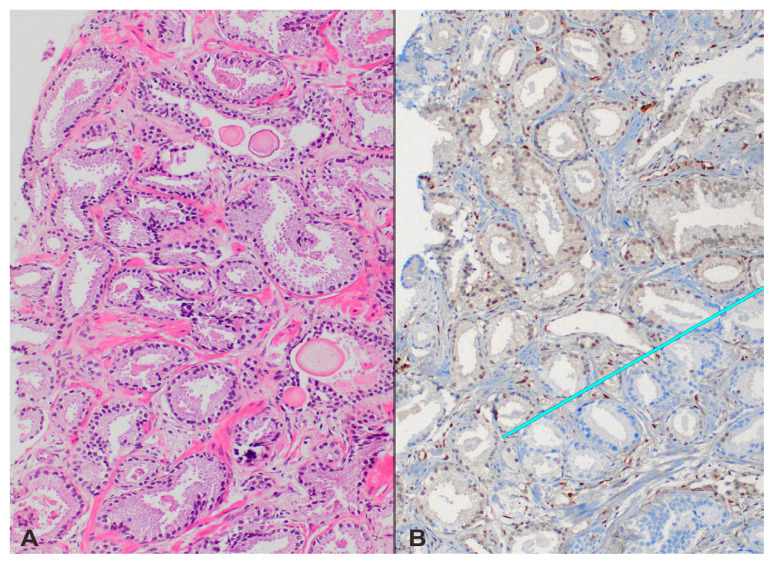
Needle biopsy with the features of GG1 prostate adenocarcinoma. H–E staining showing well-formed neoplastic glands infiltrating randomly prostatic stroma (panel A). Immunohistochemistry in this case showed heterogeneous PTEN staining (panel B); the neoplastic glands in the lower right part of the image (below the light blue line) were negative, whereas the upper ones had positive expression similar to benign epithelium (A: H–E 200×; B: Immunohistochemistry, anti-PTEN Ab 200×).

Loss of PTEN protein expression in tumors was found in 5 biopsies (15.2%) by IHC. The loss was heterogeneous, affecting 35% of cancer cells in 1 case (Figures 2A and 2B) and was total in the remaining 4. GG upgrading occurred in the RPs of all cases with PTEN loss except one (4 out of 5; 80%), whereas the upgrading frequency in patients without PTEN alteration was 42.9% (12/28). However, this difference did not appear statistically significant (p = 0.175). Rates for immunohistochemical PTEN loss were 25% and 5.9% for upgraded and nonupgraded men, respectively (p = 0.175). The tumor of the outlier case in which GG1 persisted in RP despite completely negative PTEN in the biopsy had in fact heterogeneous PTEN expression when the same stain was applied to the RP specimen, with 80% of the carcinoma cells retaining PTEN protein. Of the 5 biopsies with PTEN loss, 3 patients had PIRADS-4 and 2 had PIRADS-3 index lesions. All 3 cases with negative PTEN and PIRADS-4 index lesions were upgraded, while one out of the 2 patients with PIRADS-3 index lesions did not show upgrading in the final RP pathology. The univariate and multivariate logistic regression analyses revealed that the PIRADS score (≤3 vs. 4–5) of the index lesion and the number of involved cores in systematic biopsies (≤2 vs. >2) were independent predictors of pathological upgrading (p-values 0.024 and 0.028, respectively). PTEN loss was found not to provide a clinical guidance in this very specific problem.

No other clinicopathological variable correlated with GG upgrading after RP, including age, PSA, or PSAD (Table).

## 4. Discussion

The main goal in the management of men with PCa is to determine the patients who would benefit from active treatment of cancer and to avoid unnecessary treatment in those with clinically insignificant tumors. Although different definitions of “clinically insignificant prostate cancer (ciPCa)” have been proposed previously, it is widely accepted that GG1 disease can be classified as such and managed with active surveillance. However, it is also well established that prostate needle biopsy does not always reflect the Gleason score of the main tumor in RPs. A SEER database analysis revealed that 44% of patients with low-risk PCa (GG1, PSA < 10 ng/mL, and cT1c/2a) were upgraded and 9.7% were upstaged after radical surgery [10]. Therefore, reliable biomarkers have been continuously sought to discriminate patients who would have upper grades at RP from those in whom GG1 disease would remain. Recently, mpMRI has been highlighted as a reliable predictor for detecting clinically significant prostate cancer (csPCa) [11]. Large prospective randomized trials have consistently shown that use of mpMRI guided patient management increases the recognition of csPCa while decreasing the detection of ciPCa [12–14]. Hence, mpMRI is currently incorporated into clinical practice, being performed in all cases before prostate biopsy [1]. Studies persistently show that increasing PIRADS scores correlate with csPCa, and mpMRI appears an effective tool for predicting higher grade final pathology in cases in which the biopsy suggestion is pure GG1 disease. In the study by Nyk et al., pathological upgrading rates from biopsy to RP for PIRADS-5, PIRADS-4, and PIRADS-3 lesions were 70.5%, 62.8%, and 48.3%, respectively [15]. Our findings are similar and closely replicated the results of Nyk et al. with ratios of 75%, 66.7%, and 30% for the PIRADS scores in the same order. Therefore, any new biomarker must provide additive and independent value over mpMRI to have a clinically significant role in predicting RP grade in patients with low-risk PCa. Another parameter we found correlating with GG upgrading in our study is the number of cores with carcinoma. The multivariate logistic regression analysis reveals that core involvement of 3 or more in systematic biopsies predicts pathological upgrading independently along with a high (>3) PIRADS scored index lesion.

PTEN is a tumor suppression gene and loss of its function results in activation of the PI3K/AKT/mTOR pathway, which has been found to be associated with adverse oncological outcomes in patients with PCa [5]. Therefore, it was previously proposed as a potential biomarker to differentiate indolent disease from clinically significant prostate malignancy. Lotan et al. investigated whether PTEN status could be used to predict final RP pathology in patients with biopsy diagnosis of GG1 disease. In their study, PTEN loss was detected in 18% of upgraded men but only 7% of nonupgraded patients. The authors advocated it as an independent predictor of upgrading since tumors with PTEN loss had more risk of upgrade than those without, after adjusting for age, preoperative PSA, clinical stage, and race [7]. That study, however, was performed before the era of mpMRI. Several pathological and clinical variables along with PTEN were included in the analysis but not mpMRI-produced parameters. Similar to the results reported by Lotan et al., the rate of immunohistochemical PTEN loss in our study was higher in upgrading patients compared to those without upgrading (25% vs. 5.9%). Despite this, PTEN testing in the biopsy tissue was far from being a good biomarker for prediction of upgrading according to our results. The logistic regression analysis in our cohort did not show a correlation between PTEN status and tumor upgrading in the final pathology. We think this is mainly due to the rare occurrence of PTEN alterations in low grade PCa. Its loss is found only in a tiny proportion (15%) of our cases with GG1 pathology at biopsy. Another consideration is the intratumoral heterogeneous staining for PTEN in IHC. Thus, variable labeling of the tumor as PTEN positive or negative may occur depending on the tumor samples submitted to pathology. A tumor in one of our cases had no PTEN staining in the biopsy but was 80% positive in RP. Furthermore, solid thresholds and cut-off values for positive or negative calls have not been universally established yet. Our findings point out the importance of mpMRI and the number of biopsy cores containing tumors, rather than PTEN.

The present study has several strengths and limitations. The main strength was the standardized evaluation of pathological specimens, immunohistochemical staining, and radiological images by a very experienced team of pathologists and radiologists. As for limitations, this was a retrospective study with its inherent shortcomings. The limited sample size arising from stringent inclusion criteria and lack of power analysis to calculate optimal sample size should also be considered limitations.

In conclusion, mpMRI appears to be the best marker according to our results to foresee which GG1-PCa will be upgraded in RP. The other valuable parameter in our series that can be used in this respect is the needle core number affected by cancer. Although loss of PTEN expression is generally known to be associated with aggressive PCa, its role to predict final RP grade in cases with a biopsy diagnosis of GG1 disease has not been established yet and our findings are not supportive of its use in this way. Staining heterogeneity in PTEN immunohistochemistry is one issue that can cause conflicting results and needs further investigation for its correlation with tumor behavior.

## Figures and Tables

**Table:** The logistic regression analysis to predict upgrading in RP.

Parameters	p-value	OR	95% CI	
			Lower	Upper
**Univariate analysis**				
Age	0.466	0.968	0.886	1.057
Family history (ref. no vs. yes)	0.268	0.369	0.063	2.150
Preoperative PSA	0.437	0.930	0.776	1.116
PSA density ( ref. <0.10 vs. ≥0.10)	0.224	2.381	0.588	9.646
Biopsy technique				
• Systematic biopsy	Ref.	Ref.		
• MRI-fusion biopsy	0.812	1.269	0.179	9.021
• In-bore biopsy	0.307	4.500	0.251	80.565
Maximal tumor involvement of cores	0.235	1.015	0.990	1.041
Number of involved cores in systematic biopsy (≤2 vs. >2)^l^	0.024*	6.933	1.291	37.224
Presence of perineural invasion (ref. no vs. yes)	0.099	4.500	0.752	26.931
Laterality of tumor (ref. unilateral vs. bilateral)^l^	0.122	3.714	0.704	19.590
mpMRI index lesion PIRADS score (ref. PIRADS ≤3 vs. >3)	0.011*	7.944	1.601	39.416
mpMRI lesion multiplicity (ref. solitary vs. multiple)	0.978	1.023	0.210	4.978
Size of index lesion on mpMRI	0.182	1.094	0.959	1.248
PTEN loss by IHC (ref. no vs. yes)	0.157	5.333	0.526	54.032
**Multivariate Analysis**				
Number of involved cores in systematic biopsy (≤2 vs. >2)^l^	0.028*	19.322	1.387	269.140
mpMRI index lesion PIRADS score (ref. PIRADS ≤3 vs. >3)	0.024*	19.694	1.489	260.545
Presence of perineural invasion (ref. no vs. yes)	0.107	9.737	0.613	154.658

* Statistically significant.

l Patients who underwent in-bore biopsy were not evaluated in the analysis of laterality of tumor and the number of involved cores in systematic biopsy.

RP = Radical prostatectomy, PSA = Prostate Specific Antigen, IHC = Immunohistochemistry, mpMRI = Multiparametric Prostate Magnetic Resonance Imaging, ref = Reference, PTEN = Phosphatase and Tensin Homolog, PIRADS = Prostate Imaging-Reporting and Data System
